# Improved Detection of Paroxysmal Atrial Fibrillation Utilizing a Software-Assisted Electrocardiogram Approach

**DOI:** 10.1371/journal.pone.0089328

**Published:** 2014-02-28

**Authors:** Jürgen R. Schaefer, Dieter Leussler, Ludger Rosin, David Pittrow, Thomas Hepp

**Affiliations:** 1 Department of Internal Medicine, Cardiology, Philipps-University, Marburg, Germany; 2 Cardiology Clinic, “Kardiologie Plattform Hessen”, Marburg, Germany; 3 Medical Department, Sanofi-Aventis, Berlin, Germany; 4 Institute for Clinical Pharmacology, Medical Faculty, Technical University Dresden, Dresden, Germany; 5 Apoplex Medical Technologies GmbH, Pirmasens, Germany; University Heart Center, Germany

## Abstract

**Background:**

Automated complexity-based statistical stroke risk analysis (SRA) of electrocardiogram (ECG) recordings can be used to estimate the risk of paroxysmal atrial fibrillation (pAF). We investigated whether this method could improve the reliability of detection of patients at risk for pAF.

**Methods and Results:**

Data from 12-lead ECGs, 24-h Holter ECGs, and SRA based on separate 1-hour Holter ECG snips were collected from three groups: 70 patients with a history of pAF but who showed no AF episode in the 12-lead ECG at study entry; 19 patients with chronic AF (at study entry); and 100 young healthy individuals. AF episodes were detected by Holter ECG in 19 of the 70 non-chronic AF patients (27.1% overall, 18.6% in the first hour), and 37 of these 70 patients were classified as at risk for pAF by SRA (representing a sensitivity of 52.9% based on the first hour of analyzed recording). Fifty-four of the 70 patients also showed a sinus rhythm in the first hour. SRA detected pAF risk in 23 of these 54 patients (representing a sensitivity of 42.6%). The Holter data showed at least 1 AF episode and at least 1 hour of sinus rhythm in nine of the patients with pAF. For these patients, SRA classified 77.8% as being at risk in the first hour after the end of the AF episode, and 71.4% and 42.9% as being at risk in the second and third hours, respectively. SRA detected almost all cardiologist-confirmed AF episodes that had been recorded in 1-hour ECG snips (sensitivity, 99.2%; specificity, 99.2%).

**Conclusions:**

This outpatient study confirms previous findings that routine use of SRA could improve AF detection rates and thus may shorten the time between AF onset and initiation of prevention measures for patients at high risk for stroke.

## Introduction

Atrial fibrillation (AF) is by far the most common cardiac rhythm disorder seen in clinical practice. Currently, about 0.5–1% of the total population is affected [1], [2]. The prevalence is highly age-dependent, and climbs to 10% in 80- to 89-year-old patients [1]. Owing to lifestyle habits, and to the increased prevalence of metabolic disease and hypertension, AF diagnosis is made with increasing frequency in younger patients (“lone atrial fibrillation”, without structural heart disease) [3].

AF is associated with a 5-fold increase in stroke risk and a doubled risk of mortality [4], [5]. Since adequate anticoagulation therapy can decrease the risk of ischemic stroke in patients with AF by more than 70%, it is clear that early detection and treatment of AF is essential [6].

The current American Heart Association (AHA) and American College of Cardiology (ACC) guidelines categorize AF into three forms: paroxysmal AF (intermittent, stopping spontaneously <7 days after onset), persistent AF (lasting >7 days or terminated by pharmacological or electrical cardioversion), and permanent AF (cardioversion unsuccessful or considered of no use) [7], [8]. Most AF episodes are not noted by the patient, or they manifest with nonspecific symptoms such as fatigue or impaired exercise capacity [2]. There is very little association between clinical classification of AF (paroxysmal, persistent, or permanent) and various clinical manifestations (symptoms/lack of symptoms, different patient thresholds for seeking medical attention and subsequent rhythm documentation, and different thresholds for cardioversion attempts). There is also little association between AF persistence (time spent in AF) and these clinical manifestations. In the clinical setting, patients have been seen who have been classified as paroxysmal AF patients, with substantial time spent in AF, but who have lacked AF diagnosis due to a lack of documentation, symptoms, and/or seeking of medical attention.

The diagnosis of AF is often an incidental finding or the result of an appropriate screening examination. The definitive diagnosis is made by electrocardiogram (ECG), as a resting 12-lead ECG or as a long-term (Holter) ECG over 24 to 72 hours with automatic evaluation by appropriate software (these methods record episodes of supraventricular tachycardia). Notably, as shown in the recent UK-based screening for atrial fibrillation in the elderly (SAFE) study, many primary care professionals cannot accurately detect AF even when it is clearly present on an ECG. This indicates a need for supportive software [9]. In selected cases, e.g. after acute stroke, external or implantable event loop recorders or cardiac event recorders can also be used, over prolonged periods [10], [11]. While these devices are highly effective in detecting AF periods, a considerable proportion of them deliver false positive results, caused by supraventricular or ventricular extrasystoles and by sinus arrhythmias or sinoatrial blocks [12]. Further, if the recording is started by the patient in the presence of symptoms, the asymptomatic episodes will go undetected.

The conventional analysis of long-term ECG as the standard approach to screen for paroxysmal AF is only useful if AF occurs during the recording. Because there are often long periods without any episodes of paroxysmal AF, the search is often time-consuming, costly, and burdensome for the patient [13]. Therefore, in recent years, several algorithms for the analysis of ECG recordings have been tested (e.g., the dynamics of the RR interval, P-wave morphology, and atrial ectopy) in order to detect predictors for patients at high risk for AF or patients with undocumented past AF episodes [14].

Meanwhile, a telemedicine technology called Stroke Risk Analysis (SRAdoc, manufacturer: Apoplex Medical Technologies, Pirmasens, Germany) was introduced. According to the vendor, analysis of a 1-hour snip of a 2-channel Holter ECG can identify patients with paroxysmal AF with high reliability even if the analyzed ECG does not include any manifest AF events. SRA, based on extended Poincaré analysis [15], examines changes in heart rate dynamics that begin with the onset of an AF episode and continue despite the end of the episode, and makes use of these changes to infer the past occurrence of an unrecorded AF event [16].

This SRA software has been tested in clinical studies in different settings (a pilot study in patients with and without AF [15]; two studies in stroke patients in neurological wards [17], [18]; and the present study). The present study was the first to evaluate the software in an outpatient setting (cardiologists' offices) with typical AF patients under everyday clinical practice conditions.

We aimed to prospectively determine the sensitivity and specificity of SRA in patients with confirmed previous episodes of paroxysmal AF, but with no AF in the current ECG, and compare these parameters to those obtained for patients with manifest chronic (persistent or permanent) AF in their current ECG data. The recording of ECGs was performed by experienced cardiologists to generate data under real-life conditions.

## Methods

The study was designed as a prospective, open-label (for patients and investigators), observer-blinded (for readout of recordings), multicenter study performed in the outpatient offices of 15 research-oriented cardiologists who collaborate in the setting of the “Kardiologie Plattform Hessen” (http://www.kardiologie-plattform-hessen.de/). Healthy volunteers were recruited by the Sporthochschule Munich. The study was conducted between October 2009 and January 2010. It was purely observational, and the treating physicians – with the exception of performing a Holter ECG – were free to apply further diagnostic measures, but these were not used in this study.

The study was performed in accordance with the Declaration of Helsinki and followed general Good Clinical Practice procedures. The study protocol was approved by the local ethics committee of the Landesärztekammer Hessen. All study subjects gave written informed consent prior to participation in this trial.

### Patients with AF and healthy individuals

Individuals were suitable for inclusion if they met the following criteria:

- Patient group aged 40 years or older with confirmed paroxysmal AF: diagnosis was based on at least one 12-lead ECG recorded not more than 12 months previously, and patients were in sinus rhythm at the time of inclusion.
- Control group of patients aged 40 years or older with confirmed chronic AF (persistent or permanent AF): diagnosis was based on at least one 12-lead ECG recorded not more than 12 months previously, with manifest AF at the time of inclusion.
- Control group of individuals without AF aged 18 years or older: classification based on at least one 12-lead ECG recorded not more than 12 months previously, and absence of AF in patient history.

Exclusion criteria were as follows for all three groups: ventricular extrasystoles in patient history (>30 per hour); electrical or pharmacological conversion within the last 6 months; antiarrhythmic drugs (class I, III, or IV with the exception of verapamil); pacemaker or implanted cardioverter/defibrillator; lone AF; significant cardiac rhythm disorders. In addition, in the group without AF, exclusion criteria included supraventricular extrasystoles (>1 in 12-lead ECG upon entry) and bundle branch blocks.

### Study conduct

Investigators recorded the following clinical information on paper case report forms (CRF): demographics (age, gender), clinical data (height, weight, blood pressure, and heart rate), risk factors for AF, cardiac history, and concomitant diseases. In patients with known AF, detailed clinical information was collected (date of first diagnosis, type [paroxysmal, persistent, or permanent], diagnostic procedure, antiarrhythmic therapy, previous conversions, and therapeutic goal).

**Figure 1:**
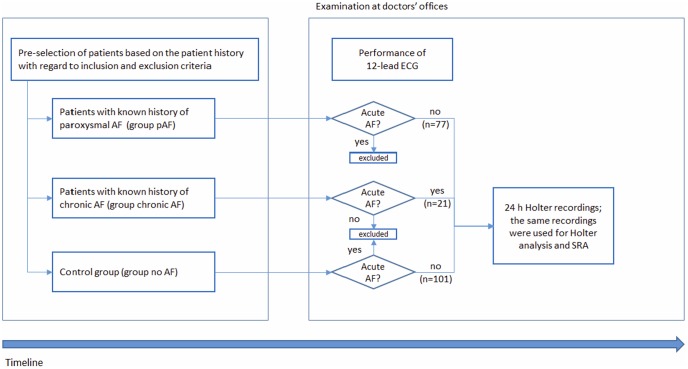
Overview of the study flow. AF =  atrial fibrillation; ECG =  electrocardiogram; SRA =  stroke risk analysis.

An initial 12-lead ECG was part of the examination to test for AF, as a component of the inclusion or exclusion criteria. This was immediately followed by Holter ECG over 20 to 24 hours as an integral part of the study (Figure 1).

All centers used the same Holter device (Lifecard CF Holter, manufactured by Spacelab Healthcare, Chesham, UK). For optimized data recording, blue sensor electrodes were used. Only light physical activity was permitted during the ECG recording. Data were recorded on one card per patient, and printouts were not necessary for the study. The data cards included a prespecified unique identifier, and the physician noted the patient number and date of recording before sending the cards to the contract research organization (CRO) for analysis.

### Analysis of ECGs

The CRO (IKKF Munich) used certified software to screen for episodes of AF (Pathfinder, manufacturer Spacelab Healthcare, Chesham, UK). Parts of the ECG marked as suspected AF by the software were independently reviewed by two experienced cardiologists in order to determine the presence or absence of AF in individual patients.

In the next step, using validated software, the complete 20–24 h Holter recording was snipped into separate 1-hour recordings and labeled by the CRO to ensure that, within the CRO, each recording could be attributed to the correct patient and time of recording. These pseudonymised 1-hour ECG snips were provided on USB sticks to Apoplex Medical Technologies and were analyzed with the SRA software at the office of the CRO. Based on the results of the software analysis, the ECG snips were categorized into four groups:

- n.a.: not analyzable (number of NN intervals too low, bad signal, and/or pathological QRS complexes);
- no risk: no pathological SRA findings (very high probability of normal sinus rhythm);
- risk of paroxysmal AF: pathological SRA findings without the presence of AF (indicative of a high probability of AF recurrence in the future);
- AF: presence of manifest AF.

To ensure blinding of the observers who were responsible for the reading of ECG snips, all information related to patients (e.g. initials, birth date, patient numbers) was deleted. This included the Spacelab metadata (hidden data) such as file identification codes, recording date and time, and identification number of the device.

Notably, the Pathfinder software for Holter ECG defines the “first hour” as the beginning of recording until the next full hour and thus is always <60 minutes. In order to achieve matching 1-hour recording snips, the offset-data for the first hour of ECG recording were used, and the last (incomplete) hour of recording was discarded. This procedure led to a minimal loss of recording time, and a maximum uncertainty of 1.7% (since the offset in the Pathfinder system is provided without seconds).

### Research questions

The study aimed to determine (1) the sensitivity of SRA for detecting patients with paroxysmal (but currently non-manifest) AF; (2) the sensitivity of SRA in patients with manifest AF (i.e., the proportion of patients in whom permanent/persistent AF was detected); (3) the specificity of SRA screening (i.e., the proportion of healthy volunteers who were correctly categorized as healthy).

### Data processing and statistical analyses

Data were extracted from three sources: clinical data as entered by the participating centers in a common database, Holter ECG data exported from the Pathfinder analysis software, and SRA data from exports of the proprietary SRA software (Apoplex Medical). Raw data sets were imported into SPSS (PASW Statistics 17.0.2 for Linux) and connected using unique identifiers. The Holter ECG data were further analyzed by SRA at the offices of the CRO, and the data were transferred electronically to IKKF for further statistical evaluation.

### SRA algorithm

The SRA algorithm consists of three major steps to calculate AF risk.

The first step is to identify QRS complexes on the ECG and to categorize them as normal or ventricular beats. To maximize accuracy, the QRS detection algorithm is based on the best two out of the three recorded leads. The classification of the beats (normal or ventricular) is based on morphology parameters like length, height, and shape of the QRS complex. Then, the RR intervals are used to calculate various, mostly nonlinear, mathematical parameters. These parameters are then used as the input for support vector machine-based classification of different groups. In the end, this results in a final classification into one of three groups: no risk, risk of paroxysmal AF, or presence of AF.

Based on this procedure, in the current study from the resulting RR list, a number of linear and (mostly) non-linear mathematical parameters were calculated in order to determine rhythm characteristics. First, principle component analysis was used to calculate the standard deviation (SD) of the minor (SD1) and major (SD2) axes of the Poincaré plots, and the ratio SD1/SD2 [19]. The dynamics of RR interval fluctuations were assessed by creating RR difference plots. Instead of plotting an RR interval against its previous one, the differences between two consecutive RR intervals were plotted and normalized by dividing them by the mean of the two corresponding RR intervals [(Ri-Ri+1)/(Ri+Ri+1). Krstacic et al. have shown that the ratio between the shortest and longest interval of the six largest consecutive RR intervals might be a predictive parameter for paroxysmal AF [20]. Since premature atrial complexes are known to play a major role in triggering AF, the number of these complexes was also included in the analysis. As proposed by Thong et al., the number of complexes that trigger the risk for paroxysmal AF depends on the preceding normal heart rate, the type of premature atrial complexes (normal complexes with sinus node reset or abnormal complexes: interpolated, full compensatory pause, or delayed sinus node reset), and also on the rhythm of the subsequent beats [21]. Thus, not all detected atrial premature complexes were rated equally, and only premature atrial complexes without sinus nodal reset were included in the analysis. Finally, regularity was analyzed by calculating the approximate entropy of the RR intervals, which is a measure of complexity in time-series analysis of ECG data [22].

Using these parameter sets, a combination of neural networks was set up. The networks were trained with data from the Massachusetts Institute of Technology (MIT) database of AF (AFDB) and the MIT-Beth Israel Hospital Normal Sinus Rhythm Database (NSRDB) [23], as well as several hundred datasets from patients with paroxysmal AF and subjects without any history of paroxysmal AF (validated by cardiologists). Together, data from these four datasets were used to classify the readouts into the described SRA categories.

Due to the mathematical nature of a neural network, it cannot explain its reasoning or how it followed a series of steps to derive the answer [24]. The results can only be confirmed by test datasets like the ones used in this study.

### Statistical analyses

For basic demographic variables and ECG results, descriptive data analysis was performed. Descriptive data are presented as means and standard deviations. SRA was compared with Holter analysis and was assessed in terms of its sensitivity (detection of AF when AF is truly present, i.e., identifying true-positives) and its specificity (recognition of absence of AF when AF is truly absent, i.e., identifying true-negatives).

## Results

### Patient disposition and characteristics

Of a total of 225 screened individuals, adequate clinical information for participation in the study was given for 199 of them. These 199 included 101 healthy volunteers, 77 patients with paroxysmal AF (but not with manifest AF at the time of the inclusion visit), and 21 patients with chronic (persistent or permanent) AF. For 15 individuals, there was no assessable Holter ECG and/or SRA, which led to exclusion of these patients from the sensitivity/specificity analyses.

Patients in the AF groups were elderly, as expected, and there were more women than men. Comorbidities were frequent in the AF groups, in particular arterial hypertension (Table 1). Healthy volunteers were on average 29±10 years old, and 67% were females.

**Table 1:** Clinical characteristics of the study groups.

	Total Subjects	Paroxysmal AF	Chronic AF*	Healthy controls
	N = 199	N = 77	N = 21	N = 101
Age, years**	49±21.9	68±9.6	70±6.9	29±10.0
Gender, % female	55.8	49.4	23.8	67.3
Body mass index, kg/m^2^ **	24.9±5.1	27.8±4.4	29.2±6.2	21.8±2.9
Blood pressure, mmHg	128/79	138/82	137/81	119/76
Heart rate per min on ECG**	71±13	65±12	82±20	73±10
**Comorbidities, %**				
Arterial hypertension	38.2	77.9	66.7	2.0
Diabetes mellitus	8.5	16.9	19.0	0
Coronary artery disease	11.6	18.2	42.9	0
Heart insufficiency	9.1	16.0	28.6	0
**Previous AF therapies, %**				
Drug conversion	18.8	22.7	4.8	n.a.
Electrical conversion	9.3	5.3	23.8	n.a.

^*^ persistent/permanent AF;

^**^Age, body mass index, and heart rate are presented as mean ± standard deviation. AF =  atrial fibrillation; n.a. =  not applicable.

### AF in the Holter ECGs

Per definition, healthy individuals had no AF signs in the Holter ECG. Patients with chronic AF had manifest AF in 16 of 19 cases (84.2%) during the complete recording time (73.7% in the first hour), while those with paroxysmal AF had manifest AF in 19 of 70 cases (27.1%) during the complete recording time (18.6% in the first hour).

Thus, for patients with paroxysmal AF (who had no AF in the 12-lead ECG at entry), the current Holter ECG had a detection rate of 27.1% during the full recording period (and 18.6% during the first hour). In three patients who had been clinically labeled as cases with chronic AF, in the Holter ECG no episode of AF was observed during the full recording (five chronic AF patients showed no AF episode in the first hour).

### Comparison of SRA vs. clinical classification after one hour of recording (70 paroxysmal and 15 chronic AF patients combined)

Of the 85 patients with paroxysmal or chronic AF, SRA identified 50 in the first hour of recording (representing a sensitivity of 58.8%), as shown in Table 2. It excluded AF in 99 of the 100 healthy individuals (representing a specificity of 99.0%).

**Table 2:** SRA vs. clinical classification (paroxysmal and chronic AF patients combined) within the first hour of recording.

		Clinical classification of paroxysmal or chronic AF by SRA		
		AF yes	AF no	Total
**SRA classification**	**AF yes**	50	1	51
	**AF no**	35	99	134
	Total	85	100	185

Sensitivity 50/85 = 0.59 Specificity 99/100 = 0.99.

SRA, Stroke Risk Analysis system; AF, atrial fibrillation.

When limiting this analysis to the 70 patients with paroxysmal AF, SRA identified 37 patients in the first hour (representing a sensitivity of 52.9%), while maintaining the specificity of 99.0% (Table 3).

**Table 3:** SRA vs. clinical classification (paroxysmal only) within the first hour of recording.

		Clinical classification of paroxysmal AF only		
		AF yes	AF no	20
**SRA classification**	**AF yes**	19	1	127
	**AF no**	28	99	147
	Total	47	100	147

Sensitivity 19/47 = 0.40 Specificity 99/100 = 0.99.

SRA, Stroke Risk Analysis system; AF, atrial fibrillation.

### Comparison of SRA vs. clinical classification in all available 1-hour snips of recording (70 paroxysmal and 15 chronic AF patients combined)

Of the 1,930 ECG readout snips from patients with clinical AF (history of AF), SRA correctly identified 1,164 of them (representing a sensitivity of 60.3%), as shown in Table 4. SRA excluded AF in 2,102 of the 2,236 snips of healthy individuals (representing a specificity of 94.0%).

**Table 4:** SRA vs. clinical classification (paroxysmal and chronic AF patients combined) using all recording snips (up to 24 hours).

		Clinical classification		
		AF yes	AF no	Total
**SRA classification**	**AF yes**	1164	134	1298
	**AF no**	766	2102	2868
	**Total**	1930	2236	4166

Sensitivity 1164/1930 = 0.60 Specificity 2102/2236 = 0.94.

SRA, Stroke Risk Analysis system; AF, atrial fibrillation.

### Comparison of SRA vs. Holter ECG in the detection of manifest AF

Five patients with chronic AF did not show any AF events in the Holter data; the analysis was performed only on snips that contained AF episodes (as confirmed by cardiologists on review of the ECGs), and all snips that contained AF episodes were analyzed. Of the 504 ECG snips from patients with clinical AF, SRA identified 500 (representing a sensitivity of 99.2%), as shown in Table 5. SRA excluded AF in 2,797 of the 2,821 ECG snips from healthy individuals (representing a specificity of 99.2%). Due to very poor signal quality, 2 out of 199 snips of the first hour could not be analyzed by SRA.

**Table 5:** SRA vs. Holter ECG in the detection of manifest AF, using all recording snips with confirmed AF episodes.

		Clinical classification		
		AF yes	AF no	Total
**SRA classification**	**AF yes**	500	24	524
	**AF no**	4	2797	2801
	Total	504	2821	3325

Sensitivity 500/504 = 0.99 specificity 2794/2821 = 0.99.

SRA, Stroke Risk Analysis system; ECG, electrocardiogram; AF, atrial fibrillation.

### Ancillary analyses

Out of the 47 patients with clinically confirmed paroxysmal AF who showed no AF on the Holter ECG (i.e., in whom Holter ECG had a detection rate of 0%), SRA indicated the presence of AF in 19 of them (representing a sensitivity of 40.4%), based on data from the first hour of recording.

SRA indicated the presence of AF in 23 of 54 patients with paroxysmal AF and sinus rhythm in the first hour of the Holter ECG (representing a sensitivity of 42.6%).

In 7 of 9 patients with paroxysmal AF and at least 1 AF episode apparent in the Holter data, but also at least 1 hour of sinus rhythm, SRA indicated the presence of AF from ECG data from the first hour after the end of the AF episode (representing a sensitivity of 77.8%). In 5 of 7 patients, SRA indicated the presence of AF from ECG data from the second hour (representing a sensitivity of 71.4%), and in 3 of 7 patients, SRA indicated the presence of AF from ECG data from the third hour (representing a sensitivity of 42.9%).

In patients with paroxysmal AF with risk factors for AF (such as hyperthyroidism or alcohol consumption), who had sinus rhythm in the first hour followed by at least one AF episode on Holter, SRA indicated the presence of AF with a sensitivity of 50.0%.

## Discussion

The detection rate of AF by intermittent rhythm monitoring strongly depends on temporal AF characteristics, and a great proportion of AF patients fail to be correctly diagnosed by this method [25]. Further, intermittent rhythm monitoring is time consuming and costly. The medical need for detecting paroxysmal AF and the high uncertainty of intermittent rhythm monitoring justify the search for better tools to detect AF.

The present study, performed under everyday clinical conditions, indicates that, compared with Holter ECG, SRA is advantageous in that it is able to predict AF in a patient who does not have AF at the time of recording; SRA had a 52.9% sensitivity and 99.0% specificity for indicating the presence of paroxysmal AF from data collected during the first hour of recording. When assessing all recording periods, SRA detected AF with a sensitivity of 99.2% and a specificity of 99.2%, and thus was virtually equivalent to Holter ECG. The latter did not have significant benefits with respect to the detection of AF (in 1-hour snips with manifest AF episodes), despite the fact that they were evaluated with more time and scrutiny compared with “real-life” (everyday clinical) scenarios. Overall, this supports the usefulness of SRA in effective screening for paroxysmal AF, as other methods such as the use of event recorders are time-consuming and costly, making them unsuitable for screening on a large scale.

According to the ancillary analyses, the ability to detect AF by SRA seems dependent on the time point of emerging AF episodes; it decreases with longer periods in sinus rhythm. In our study, sensitivity ranged from 40% (no AF episodes during Holter ECG) to 78% when assessing the first 1-hour snip after an AF episode. It is unclear whether the increased sensitivity in risk groups is due to the increased risk and/or the presence of at least one AF episode outside the first 1-hour snip.

To date, data on the clinical usefulness of SRA have been reported from three studies.

The first was a pilot study that derived the optimal analysis algorithm and retrospectively validated it. SRA of 29 patients with paroxysmal AF, 9 patients with chronic AF, and 21 healthy controls predicted paroxysmal AF with a sensitivity of 89.7% (26 of 29) and a specificity of 81.0% (17 of 21), whereas conventional long-term ECG analysis had a sensitivity and specificity of 13.8% (4 of 29) and 100% (21 of 21). The sensitivity of SRA for detecting AF in paroxysmal AF patients during time periods lacking episodes of fibrillation was 47.5% (258 of 543 1-hour snips) with a specificity of 96.7% (379 of 392 hours) [15].

The second study assessed patients who were in a neurological ward after suffering from stroke or transient ischemic attack (TIA). This study sought to determine whether a 1- to 2-hour SRA assessment could identify paroxysmal AF. Paroxysmal AF was newly diagnosed in 29 (21.3%) of 136 enrolled patients by continuous bedside ECG monitoring. The sensitivity of SRA for indicating the presence of paroxysmal AF in these high-risk patients, compared with continuous bedside ECG monitoring, was 72%. Specificity in the same patients was 63%, while in 24-hour Holter monitoring, episodes of AF were observed in only 23% [17].

The third study, a recent prospective study by Rizos et al., evaluated the effectiveness of detecting paroxysmal AF after stroke. Out of 496 patients in the stroke units participating in the study, 68 had newly detected AF. Of these, 27 had persistent AF and 41 had paroxysmal AF, as documented by at least one method, as follows. Holter ECG (median duration 24 hours), continuous ECG monitoring (median duration 64 hours), and SRA applied on the continuous ECG data detected paroxysmal AF in 34.1%, 65.9%, and 92.7% of the patients, respectively (p<0.001 SRA versus continuous ECG) [18]. SRA failed to detect paroxysmal AF in 3 of 41 patients, and falsely detected AF in 14 cases. In patients with persistent AF, SRA correctly identified 100% of the cases. As a consequence of AF detection, 76% of the newly diagnosed patients received oral anticoagulants for repeat stroke prevention [18].

Some methodological considerations have to be taken into account for the assessment of the clinical usefulness of our study. Since we wanted to make certain that the control group did not include any patients with AF, we decided to test this system in a control population that could with high certainty be presumed to be healthy; namely, sports students. As a consequence, our healthy controls were significantly younger, so that we have no age-matched control group. However, the detection of AF should not be influenced by this fact. Among the strengths of the study are the multicenter setting, the analysis of typical 24-hour Holter recordings, and the focus on the first 1-hour snip, which all represent common clinical practice. While the study of Rizos et al. applied different observation times for Holter and continuous monitoring (which introduces an obvious bias in terms of the chances for AF detection) [18], our study used the same time periods for the Holter ECG and the SRA device. Further, by investigating typical Holter ECG recording times of up to 24 hours, clinical practice is represented. It has been reported that the routine 24-h Holter ECG detects about 5% of new paroxysmal AF cases, and that the detection rate can be increased to 12.5% by applying Holter ECG over 7 days in patients with stroke [26], [27]. However, such extended recording times are often not feasible or too expensive in the clinical practice setting.

In conclusion, based on our study results, a 1-hour SRA analysis – supplemented by one or more 24-hour SRA analyses in cases of suspected paroxysmal AF – could substantially improve the detection of AF in patients with hitherto unknown AF. False-positive findings must be considered and excluded by expert review of the 12-lead or Holter ECG. The routine use of SRA cannot replace the established diagnostic measures nor is it the basis for far-reaching decisions regarding therapy with considerable side effects. However, our results, in accordance with results from previous studies, indicate that SRA does increase the diagnostic value of ECG recording for the detection of paroxysmal AF. The approach permits more careful and sensitive detection of AF, at least in those patients who would have been missed using only a conventional Holter ECG approach (roughly 50%). Thus, SRA may facilitate early AF diagnosis, with the possibility of the initiation of improved and timely prevention measures in patients at high risk for stroke. Notably, the relationship between stroke and time in AF is evasive. There is little or no evidence showing a reliable cutoff between time spent in AF and the risk of stroke [28], [29].

Future studies with substantially more patients could allow several subgroup analyses. This would allow investigation of SRA and various parameters of interest, including age, presence of other known rhythm disorders, drugs, AF burden, time of last onset of AF, or time of next onset of AF.
